# Stormwater runoff driven phosphorus transport in an urban residential catchment: Implications for protecting water quality in urban watersheds

**DOI:** 10.1038/s41598-018-29857-x

**Published:** 2018-08-03

**Authors:** Yun-Ya Yang, Gurpal S. Toor

**Affiliations:** Department of Environmental Science and Technology, University of Maryland, College Park, MD 20742 USA

## Abstract

Increased stormwater runoff in urban watersheds is a leading cause of nonpoint phosphorus (P) pollution. We investigated the concentrations, forms, and temporal trends of P in stormwater runoff from a residential catchment (31 low-density residential homes; 0.11 km^2^ drainage area) in Florida. Unfiltered runoff samples were collected at 5 min intervals over 29 storm events with an autosampler installed at the stormwater outflow pipe. Mean concentrations of orthophosphate (PO_4_–P) were 0.18 ± 0.065 mg/L and total P (TP) were  0.28 ± 0.062 mg/L in all runoff samples. The PO_4_–P was the dominant form in >90% of storm events and other–P (combination of organic P and particulate P) was dominant after a longer antecedent dry period. We hypothesize that in the stormwater runoff, PO_4_–P likely originated from soluble and desorbed pool of eroded soil and other–P likely originated from decomposing plant materials i.e. leaves and grass clippings and eroded soil. We found that the runoff was co-limited with nitrogen (N) and P in 34% of storm events and only N limited in 66% of storm events, implicating that management strategies focusing on curtailing both P and N transport would be more effective than focussing on only N or P in protecting water quality in residential catchments.

## Introduction

Urbanization influences the structure, function, and dynamics of landscape, which, in turn, increases the release and transport of pollutants from land to water^1–3^ and leads to environmental and water quality impacts^4,5^. Stormwater runoff is a major cause of physical, chemical (i.e. nutrients), and microbial degradation of receiving waters^6,7^. Nitrogen (N) and phosphorus (P) are of particular concern and interest in urban stormwater runoff due to their role in eutrophication of water bodies, onset of harmful algal blooms, and fish kills^8,9^. Consequently, a better understanding of N and P in stormwater runoff in urban areas and the relative contributions of N and P to stormwater runoff is needed for effective stormwater management.

Land use change, particularly the impervious surfaces, not only alter urban hydrology but have implications for biogeochemical transformations within the watersheds^10^. In studies conducted in 85 coastal watersheds in Texas and Florida, impervious areas were found to considerablly affect the hydrologic dynamics i.e. stream flow^11^. Further, a positive relationship was observed between nutrient export and urbanization, with increase in impervious area increased P export from 24.5 to 83.7 kg/km^2^/yr in a small urban watershed in Maryland^3^. Loading of N and P into urban areas is increased by importing natural (i.e. atmospheric deposition), anthropogenic (i.e. fertilizers, pet waste, automotive detergents), and biogenic materials (i.e. lawn clippings and leaves)^12–16^. Source tracking studies in urban waters have shown that atmospheric deposition^17,18^, lawn fertilizers^15,18^, landscape irrigation^19^, and sewage^20^ can contribute significant N to waterways. The linkages between urbanization and increased N and P export is well established; however, the contributions and dynamics of N and P are often site-specific and have not yet been activity investigated in growing residential developments in the nation.

The synchronicity between N and P in aquatic environment has been widely used as an ecological indicator of biological growth and nutrient limitation^21–24^. Previous studies have shown N-limitation in the coastal ecosystems and suggested that excessive N loading can substantially affect coastal phytoplankton communities^25,26^. Whereas, others have observed P-limitation in warm-temperate and tropical estuaries that have elevated N concentrations^27–29^. Using a meta-analysis, Elser *et al*.^30^ reported that co-limitation by N and P occurs frequently in freshwater, marine, and terrestrial ecosystems. Seasonal shifts from P limitation in spring to N limitation in summer were observed in estuaries systems^31^. Considering the vital role of urban stormwater runoff in regulating the nutrient cycles in watersheds^32,33^, it is critical to characterize the nutrient concentration and molar ratio of total N to total P (TN:TP) in the stormwater runoff to preserve the ecological functions and water quality in downstream water bodies.

In regions with growing urban population, strategies to reduce impacts of nutrients on urban waters are essential. The population in the state of Florida increased from 12 million to 21 million over the past three decades^34^. In addition, high rainfall (~137 cm per year), presence of sandy soils, and high groundwater tables in Florida are ideal conditions that potentially can deliver a significant amount of nutrients to surface waters^35^. To date, numerous studies have been carried out to characterize stormwater runoff and nutrient export from urban watersheds^15,36,37^. However, the potential contribution of nutrients carried in the stormwater runoff from urban residential neighborhoods (hereafter referred to as residential catchments) in subtropical areas has not been intensively studied. Thus, we asked the following research questions: (*i*) What are the concentrations and dominant forms of P in residential stormwater runoff? (*ii*) What temporal variability is manifest in the P composition in stormwater runoff?, and (*iii*) What are the potential limiting nutrients in residential stormwater runoff? This study was designed to fill the knowledge gaps on P forms in stormwater runoff generated in a low-density residential catchment, where samples were collected over five months that covered a wet season. An improved understanding of how dynamics of P forms and limitation of nutrients in urban residential stormwater runoff are affected by seasonal rainfall may help residential land developers, urban planners, and policy makers devise science-based management strategies for protecting water resources.

## Results and Discussion

### Overview of phosphorus forms in rainfall and stormwater runoff

**Figure 1:**
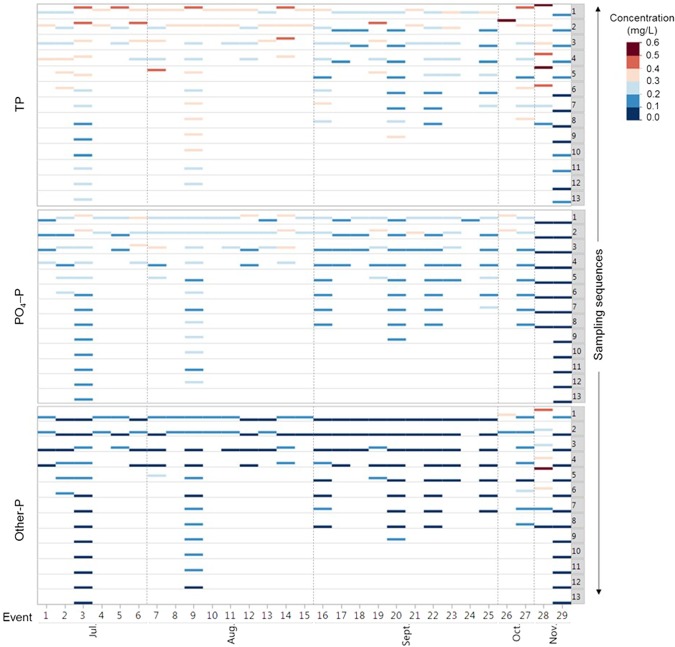
Heatmap showing concentration of TP, PO_4_–P, and other–P in the stormwater runoff (n = 153) from 29 individual storm events during July–November 2014 with sampling sequences 1 to 13, which refer to individual samples collected in 5-minute intervals during each event.

**Figure 2:**
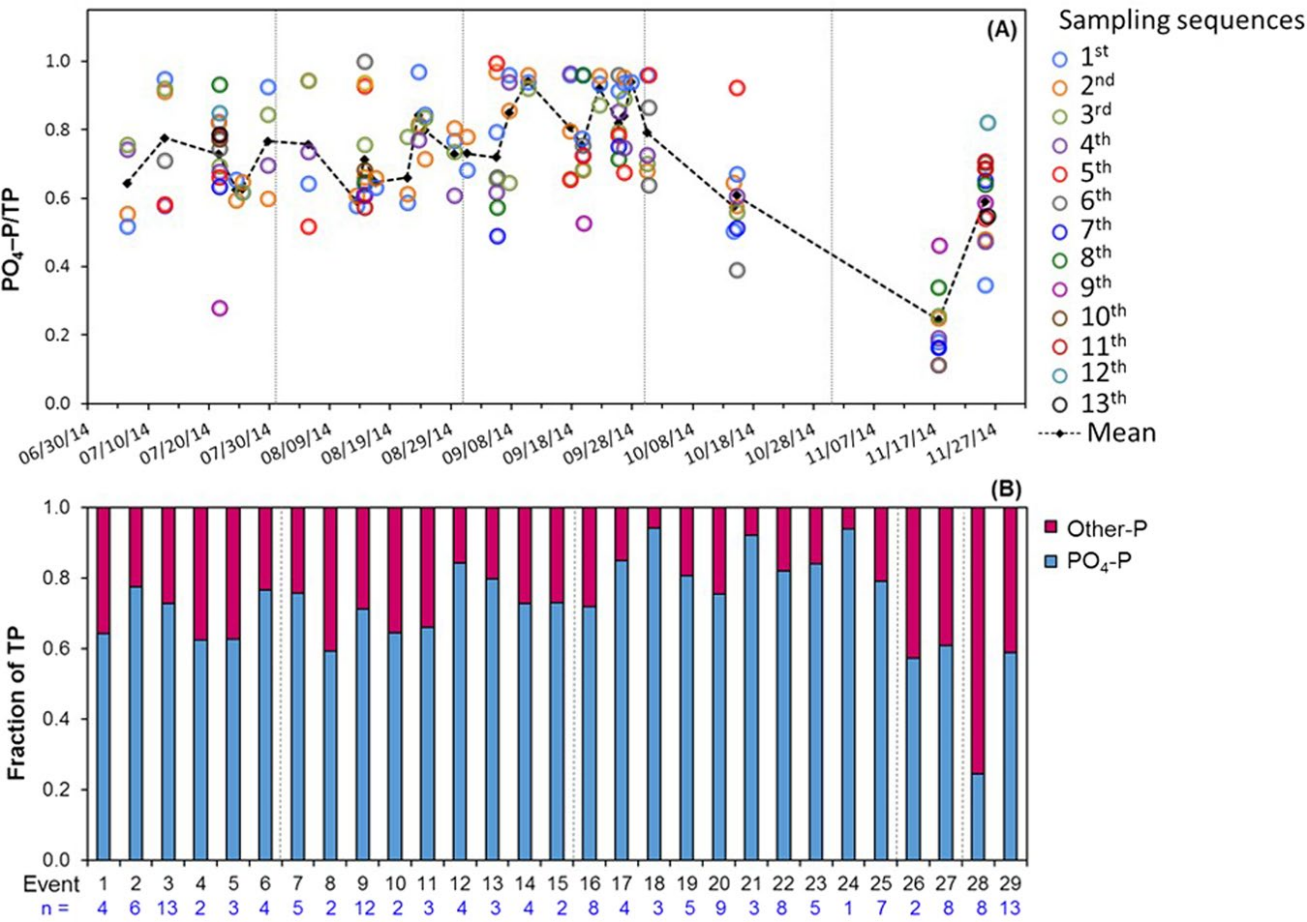
(**A**) Proportion of PO_4_–P to TP in stormwater runoff (n = 153) with sampling sequences 1 to 13, which refer to individual samples collected in 5-minute intervals during each event. The dashed line shows change of mean values from individual events. (**B**) Fraction of PO_4_–P and other–P in residential stormwater runoff over 29 sampling events (n = 153). Event 1–6, 7–15, 16–25, 26–27, and 28–29 occurred in July, August, September, October, and November, respectively. Numbers in blue are total number of sequential 5-minute samples collected during each event.

In the rainfall, mean (July–November; n = 22) TP, PO_4_–P, and other–P concentrations were 0.24 mg/L, 0.09 mg/L, and 0.15 mg/L, respectively (Table 1). Further, mean other–P:TP (0.63) was greater than mean PO_4_–P:TP (0.37) in rainfall samples. In stormwater runoff, mean (July–November; n = 153) TP, PO_4_–P, and other–P concentrations were 0.28, 0.18, and  0.10 mg/L (Table 1, Fig. 1). Of 29 sampling events, over 90% of stormwater runoff samples had PO_4_–P:TP greater than 0.50 in the low-density residential catchment, whereas the remainder of the runoff samples, collected during the later sampling events (events 28 and 29), had other–P as the dominant form (Fig. 2). Other–P was the dominant form in the rainfall and PO_4_–P was the dominant form in the stormwater runoff as also observed in our previous study of medium- and high- density residential catchments in the area^17^.

**Table 1:** Concentrations of phosphorus in rainfall and stormwater runoff collected from individual 29 storm events during July–November 2014.

Event number (Sampling date)	Number of runoff samples	TP (mg/L)		PO_4_–P (mg/L)		Other–P	
		Rainfall	Stormwater runoff	Rainfall	Stormwater runoff	Rainfall	Stormwater runoff
1 (7/6/14)	4	0.20 ± 0.112^a^	0.27	0.08 ± 0.063^a^	0.17	0.14 ± 0.071^a^	0.10
2 (7/12/14)	6		0.28		0.21		0.07
3 (7/21/14)	13		0.27		0.21		0.06
4 (7/24/14)	2		0.36		0.22		0.13
5 (7/25/14)	3		0.29		0.18		0.11
6 (7/29/14)	4		0.35		0.27		0.08
1–6 (Jul.)	total: 32		0.29 ± 0.080^a^		0.21 ± 0.060^a^		0.08 ± 0.046^a^
7 (8/5/14)	5	0.27 ± 0.032^a^	0.32	0.08 ± 0.023^a^	0.24	0.16 ± 0.067^a^	0.08
8 (8/13/14)	2		0.38		0.22		0.15
9 (8/14/14)	12		0.31		0.22		0.09
10 (8/16/14)	2		0.35		0.23		0.12
11 (8/21/14)	3		0.32		0.21		0.11
12 (8/23/14)	4		0.26		0.22		0.04
13 (8/24/14)	3		0.29		0.23		0.06
14 (8/29/14)	4		0.39		0.29		0.10
15 (8/31/14)	2		0.35		0.26		0.09
7–15 (Aug.)	total: 37		0.32 ± 0.058^a^		0.23 ± 0.045^a^		0.09 ± 0.051^a^
16 (9/5/14)	8	0.20 ± 0.108^a^	0.25	0.12 ± 0.093^a^	0.17	0.10 ± 0.065^a^	0.07
17 (9/7/14)	4		0.20		0.15		0.05
18 (9/10/14)	3		0.21		0.19		0.02
19 (9/17/14)	5		0.33		0.26		0.08
20 (9/19/14)	9		0.19		0.14		0.05
21 (9/22/14)	3		0.28		0.26		0.02
22 (9/25/14)	8		0.21		0.17		0.04
23 (9/26/14)	5		0.25		0.22		0.04
24 (9/27/14)	1		0.24		0.14		0.09
25 (9/30/14)	7		0.22		0.18		0.04
16–25 (Sept.)	total: 53		0.23 ± 0.060^a^		0.18 ± 0.058^a^		0.05 ± 0.036^a^
26 (10/14/14)	2	0.23 ± 0.114^a^	0.56	0.07 ± 0.036^a^	0.32	0.15 ± 0.084^a^	0.24
27 (10/15/14)	8		0.31		0.18		0.13
26–27 (Oct.)	total: 10		0.36 ± 0.125^a^		0.21 ± 0.068^a^		0.15 ± 0.076^a^
28 (11/17/14)	8	0.28 ± 0.035^a^	0.37	0.10 ± 0.021^a^	0.08	0.18 ± 0.014^a^	0.29
29 (11/25/14)	13		0.11		0.06		0.05
28–29 (Nov.)	total: 21		0.21 ± 0.159^a^		0.07 ± 0.011^a^		0.14 ± 0.152^a^
1–29 (Jul.–Nov.)	total: 153	0.24 ± 0.036^b^	0.28 ± 0.062^b^	0.09 ± 0.018^b^	0.18 ± 0.065^b^	0.15 ± 0.028^b^	0.10 ± 0.042^b^

^a^Monthly mean value ± standard deviation. ^b^Seasonal mean value ± standard deviation.

### Temporal variability of phosphorus concentrations and forms

**Figure 3:**
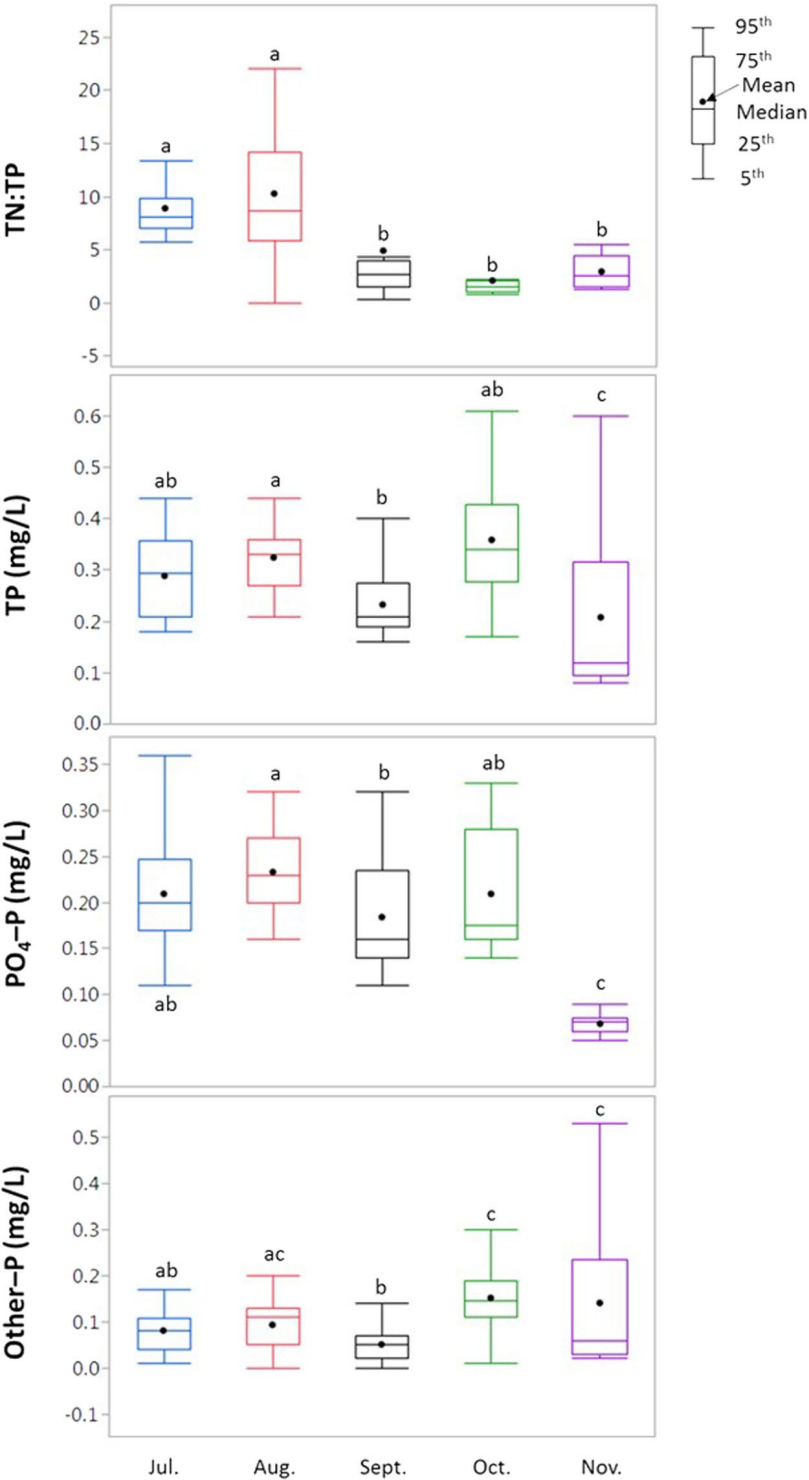
The molar ratio of TN:TP and concentration distribution of P forms in stormwater runoff (n = 153) during July–November 2014. The different letters indicate significant differences among months (AVOVA; *p* < 0.05).

Concentrations of TP, PO_4_–P, and other–P were variable throughout the sampling events and displayed no temporal trends in 29 individual storm events (Fig. 1, Supplementary Figs S1 and S2). Concentrations of PO_4_–P and TP in stormwater runoff were significantly (*p* < 0.05) greater in July, August, September, and October than November (Fig. 3). In contrast, concentrations of other–P were significantly (*p* < 0.05) greater in November than other sampling months. Several hydrological factors such as antecedent rainfall characteristics, and flow connectivity between P sources and receiving waters can affect temporal dynamics of P^38^. A positive correlation (*r*^2^ = 0.77) between other–P and antecedent dry period was observed in this study (Supplementary Fig. S3). Greater concentration of other–P in November was likely due to the longer antecedent dry period (34 days). This finding is consistent with the previous study conducted by Chew *et al*.^39^ who also observed that a greater P build-up in urban road surfaces is associated with the longer antecedent dry period. In arid urban watersheds, precipitation volume and impervious surface were found to have great influence on both N and P exports in stormwater runoff^38^. Further, the greater nutrient export in stormwater runoff often occur during the high-flow season in a year^40^. Researchers have also suggested that more intense and frequent rainfall during the beginning of rainfall season can wash-off greater amount of particulates^41^ causing greater leaching of dissolved P from sediments or organic materials as compared to storm events later in the season. Our data supports this observation. Overall, the results suggest that P sources in the stormwater runoff changed over the storm events during the season.

The variation in the temporal concentrations during storm events implies that P in the stormwater runoff was contributed by mixing of multiple sources from the residential catchment. The potential P sources in urban catchments include atmospheric deposition, lawn fertilizers, and P transport from soil, particulate matter such as plant debris, and pet waste. Studies have shown that particles carried by wind are a major (90%) contributor of atmospherically deposited P^42,43^. The P forms in stormwater runoff were not correlated with P in rainfall (data not shown), which suggests that P sources in stormwater runoff were likely different from rainfall. Further, lawn fertilizers are unlikely to be the source of P in our stormwater runoff as most Florida soil are naturally high in P^44^ and P fertilizers are not recommended in our study area. This low-density residential catchment had 61% pervious area (occupied by tree canopy and lawns), thus, P mineralization from degradation of organic materials such as lawn grass clippings and tree leaves and P desorption and dissolution from soil (eroded sediments) likely contributed P in the stormwater runoff, as this was also observed in our previous study of medium- and high- density residential catchments^17^, urban stormwater^45^, and other urban watersheds^15,46^.

Within 29 individual storm events from July to November, the PO_4_–P:TP in the stormwater runoff was highly variable and ranged between 0.1 and 1. The mean PO_4_–P:TP increased from July (0.72), August (0.73) to September (0.81) and then decreased from October (0.60) to November (0.46) (Fig. 2). The decreasing progression of PO_4_–P:TP in the last two months is attributed to the changes in P sources as continuous rainfall during the first three months likely exhausted terrestrial PO_4_–P sources. During the late storm events (October–November), there were greater opportunities for eroded sediments (due to landscaping practices such as edging and longer antecedent dry period) that contain particulate P (which is part of the other–P) to accumulate on impervious surfaces, which were subsequently washed away with rainfall to stormwater runoff. As a result, other–P was the predominant form in the late storm events (events 28–29), with mean concentrations that were two-folds significantly (*p* < 0.05) higher (n = 21; 0.14 mg/L) than previous storm events (events 1–27; n = 132; 0.07 mg/L) (Fig. 3). This illustrates that P associated with plant debris and the fine fraction of soil sediment was transported in the stormwater runoff. Data suggest that the plant debris and eroded sediments in residential catchments should be removed quickly as rainfall can extract nutrients from the debris and contribute P in stormwater runoff^45^.

### Stoichiometric controls on nitrogen and phosphorus in stormwater runoff

**Figure 4:**
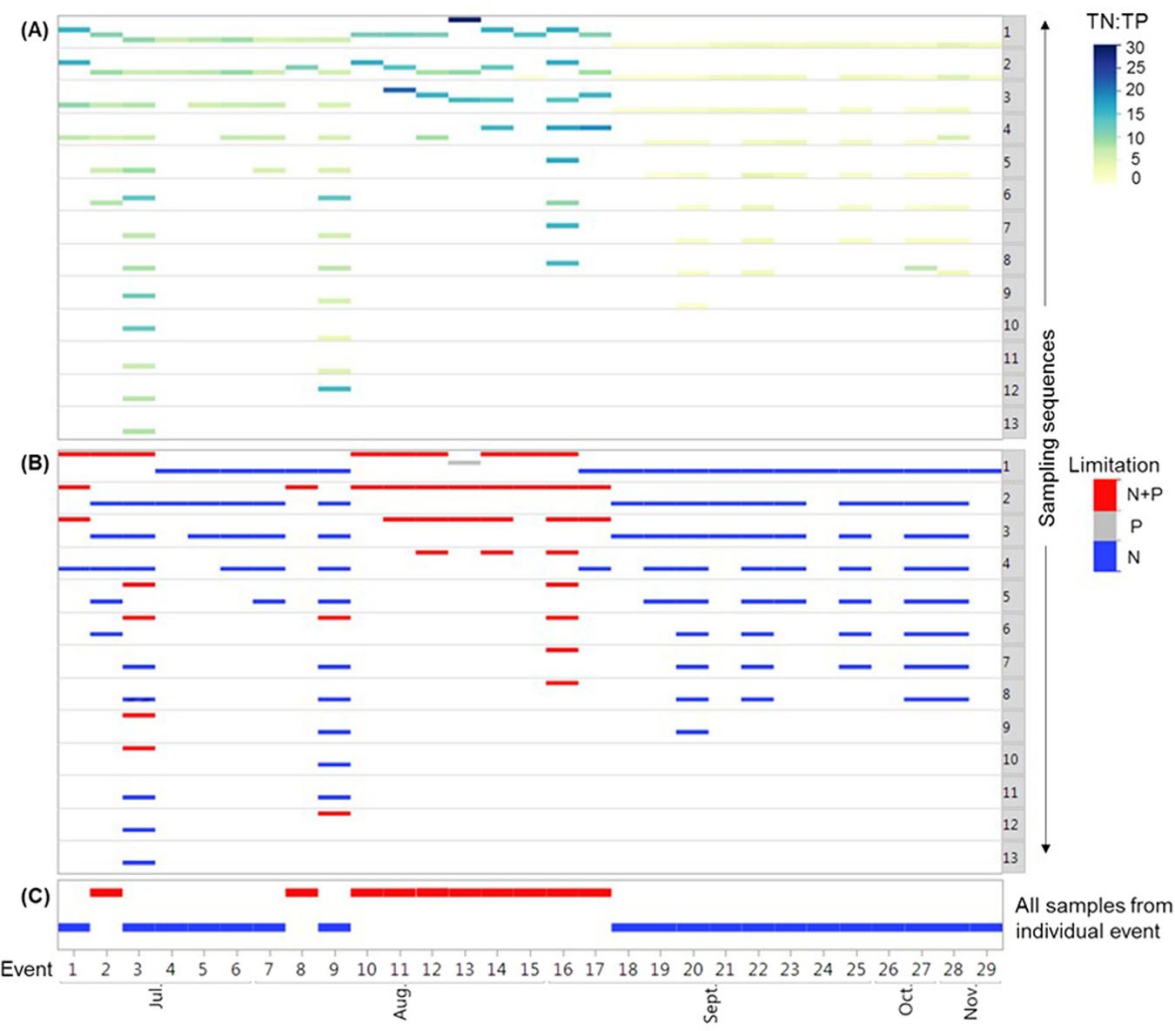
Heatmap showing (**A**) TN:TP, (**B**) N and P limitation in stormwater runoff (n = 153) from 29 individual events during July–November 2014 with sampling sequences 1 to 13, which refer to individual samples collected in 5 minute intervals during each event, and (**C**) mean event N and P limitation. Strict P limitation (TN:TP ≥ 23 by mass), strict N limitation (TN:TP ≤ 9), or N and P co-limitation (23 > TN:TP > 9), as described by Guildford and Hecky^22^ and Paerl *et al*.^47^.

Nutrients from multiple potential sources in urban residential areas are transported via stormwater runoff into the receiving waters (e.g., lakes, rivers, coastal waters). The dual controls on N and P are important in the management of watershed scale nutrient sources to reduce the eutrophication as both N and P limit the phytoplankton growth and productivity in the aquatic systems^47^. The molar ratio of TN:TP has gained worldwide acceptance in the aquatic and terrestrial systems as an indicator of phytoplankton growth and nutrient cycling^48,49^. To understand potential nutrient limitation and abundance in residential stormwater runoff, we compared the patterns of TN:TP molar ratio using the stoichiometric threshold derived from global patterns of phytoplankton stoichiometry as described by Guildford and Hecky^22^. Strict N or P limitation is suggested when the mass TN:TP is smaller than 9 or larger than 23, respectively. Systems with TN:TP between 9 and 23 indicate N and P co-limitation^22^. The molar ratio of TN:TP in individual stormwater runoff samples (n = 153) varied from as low as 0.3 to as high as 29.3 (Fig. 4A). Of 29 storm events, 19 events had mean TN:TP of less than 9 indicating potential N limitation and 10 events had mean TN:TP between 9 and 23 which indicates potential N and P co-limitation (Fig. 4B,C). More specifically, storm events co-limited by N and P were observed mostly in August (Fig. 4C). In our previous study of six medium- and high- density residential catchments^17^, storm events co-limited by N and P were observed in all six catchments, while N limitation was only observed in two catchments (Fig. S4). The relative contribution and limitation by N and P varied across different types of residential catchments likely due to the urban heterogeneity (i.e. residential development patterns). Overall, the difference in nutrient limitations and abundances between stormwater runoff events over the season suggests different transport behavior of N and P in stormwater runoff.

Concentrations of TP were not statistical different (*p* > 0.05) in stormwater runoff events during July – October (Fig. 3) implying that due to the P–rich geology in the region (our study catchment), wash-off of TP with rainfall is not source limited. In other words, there is plenty of P available in the study catchment where the rainfall–runoff dictates the net amount of TP that can be lost in stormwater runoff. In contrast, concentrations of TN were significantly (*p* < 0.05) higher in the stormwater runoff events that occurred in the active wet season (mean: 0.97 mg/L)^18^ as compared to later in the season (mean: 0.34 mg/L). This suggests that high-intensity rainfall events during the wet season caused greater wash-off of N from various sources (e.g., atmospheric deposition, N fertilizers) as season progressed. Studies have shown that atmospheric N deposition can be a significant contributor of N in urban water systems^20,50,51^. Our previous studies conducted in this low-density residential catchment and other six medium- to high- density residential found that more than 50% of NO_3_–N in urban stormwater runoff during the wet season (i.e. July–September) originated from atmospheric deposition^17,18^. Therefore, it could be implied that high TN:TP in stormwater runoff during the wet season was due to the increasing amounts of N inputs from atmospheric sources. In contrast, stormwater runoff was more N–limited during storm events that occurred in October and November due to less atmospheric N deposition. This observation supports data from a study that observed shifting of nutrient limitation in lakes and further concluded that atmospheric N deposition increased the stoichiometric ratio of N and P in lakes across three geographic regions (i.e. Norway, Sweden, and the U.S.)^52^. In a previous study in forested watersheds, researchers also found higher N:P during storm events as compared to the base flow conditions likely due to the increased atmospheric N deposition and extreme storm events^53^.

Controlling eutrophication of coastal waters requires careful basin-specific management practices for both N and P, and reducing N and P in stormwater runoff from urban residential catchments is the first and foremost opportunity to reduce terrestrial nutrient inputs. Numerous studies have stated that it is often not possible to effectively control nutrient pollution in aquatic ecosystems by controlling only a single nutrient^42,54^ due to the highly variable nature of nutrient limitation^55^, the diversity of nutritional needs among aquatic organisms^54,56–59^, and the shifting nutrient problems to downstream ecosystems^47^. In many circumstances, one nutrient may be scarce during a particular period and different species of aquatic organisms may be simultaneously limited by different nutrients^54,57,59^. In addition, the potential harmful effect of nutrient concentrations in streams may not be observed until some distance downstream. Controlling only one single nutrient could also cause another to become limiting when the limitation thresholds of N and P are close in an ecosystem^54^. Thus, it has been suggested that controlling both N and P provides the greatest likehood of nutrient pollution management and protecting aquatic ecosystems^42^. In summary, we suggest that a better understanding of how stormwater runoff and P loss mechanisms interact to influence nutrient limitations in urban residential runoff across different geographic regions and climatic zones is needed to develop effective management strategies to reduce P and N delivery to the coastal water bodies.

### Implications for phosphorus management in residential catchments

The combined effect of N and P enrichment in water bodies has resulted in accelerating eutrophication and leading to severity of harmful algal bloom across the world^60–62^. Harmful algal blooms in Florida coastal waters are an ongoing problem^63^. Therefore, there is a potential for urban water systems to cause adverse downstream impacts if nutrients in stormwater runoff are not adequately attenuated before the runoff makes its way to receiving water bodies. For example, mean concentrations of TP (0.11–0.56 mg/L) in all 29 stormwater events exceeded threshold TP limit of 0.1 mg/L in flowing waters for eutrophication (Table 1 and Fig. 3, Supplementary Figs S1 and S2)^64^. It has been shown that the most common and most biologically available P form i.e. PO_4_–P has the greatest impact on water bodies^8^. We found that PO_4_–P was the dominant P form over 90% of stormwater runoff events (Fig. 2), which further highlights the importance of curtailing PO_4_–P loss from urban land to water bodies. The variable concentrations of PO_4_–P in most storm events and higher other–P later in the season suggests that different management strategies are needed to control P loss in residential catchments. For example, the reduction in the amount of surface runoff and the improvement in the quality of stormwater before runoff waters reach water bodies have been suggested as the main management practice^65^. As the removal mechanism of PO_4_–P is via adsorption onto the media during transport^66^; one approach that could facilitate PO_4_–P removal is using green infrastructure that would allow P to infiltrate into soil, thus, reducing P concentrations in stormwater runoff. The effects of street sweeping as a nutrient management practice in urban areas have been investigated in previous studies^67^. A recent study by Selbig^45^ found that significant reduction (71–84%) in the total and dissolved forms of P and N loads can be achieved if the leaf litter accumulated on streets is removed prior to the rainfall. Thus, reduction in other–P (the predominant form after a long antecedent dry period) in stormwater runoff from impervious surface could be accomplished by street cleaning on a regular basis. Further, better landscape designs and management practices in residential catchments may assist in reducing the nutrient losses in stormwater runoff and reducing the environmental impacts of polluted runoff waters in the urban waters.

## Conclusions

The combination of land development, rainfall characteristics, and the time interval between rainfall events affect the quantity of stormwater runoff and amount of pollutants in urban waters. Understanding P dynamics in stormwater runoff can help to implement and enhance the effectiveness of strategies to control P loss and transport to receiving waters. This study investigated the concentrations, forms, and temporal trends of P in stormwater runoff in a low-density residential catchment over five months of sampling (29 storm events). We also compared stoichiometric controls on nutrients in stormwater runoff across different types of residential catchments. The concentrations of TP (0.11–0.56 mg/L) in individual stormwater events were greater than the critical TP level of 0.1 mg/L of eutrophication for surface waters. Further, PO_4_–P was the dominant form (>57% of TP) in more than 90% of stormwater runoff events indicating immediately bioavailablity of P in downstream water bodies. The TN:TP molar ratios indicated that stormwater runoff was co-limited with N and P in 34% of storm events and N was limited in 66% of storm events, thus, implying that management strategies that focus on reducing both N and P inputs would be more effective to protect and preserve water quality in receiving urban water bodies. When comparing different types of residential catchments, the relative limitation by N and P varied across low-, medium-, and high- residential catchments, which suggests that urban heterogeneity (i.e. residential development patterns) resulted in different mechanistic controls on N and P transport in stormwater. The findings from this work enable us to better understand the effects of residential stormwater runoff on urban water quality and may help to develop strategies to reduce the adverse impacts of land development on receiving urban waters.

## Materials and Methods

### Study site

The detailed description of the study site can be found in Yang and Toor^18^. In brief, the study site is classified as a low-density residential catchment, which consisted of 31 single-family homes (average home size: 409 m^2^) and located in Tampa Bay, Florida (Supplementary Fig. S5). Total area of the residential catchment including a connected stormwater pond was 0.11 km^2^. Of total area, 61% area was pervious that contained 29% area occupied by St. Augustine turfgrass (*Stenotaphrum secundatum*) lawns and 32% occupied by tree canopies of live oak (*Quercus virginiana*), whereas 37% area was impervious that consisted of rooftops, patios, driveways, sidewalks, and roads, and 2% was occupied by stormwater pond. The study site has subtropical climate with 2014 average monthly annual air temperature of 14–27 °C. The monthly rainfall was higher in wet season months (240 mm in July, 140 mm in August, and 340 mm in September) and lower immediately after the wet season (6 cm in October and 18 cm in November)^68^. In comparison, the average annual rainfall during the last 10 years (2004–2014) in the area was 130 cm (range: 94–153 cm), of which 58% occurred during the wet season (June–September; Supplementary Figs S6 and S7).

### Sample collection and analysis

The unfiltered stormwater runoff samples were collected from the end of the outlet pipe that drains residential catchment. An ISCO Avalanche 6712 refrigerated autosampler (Teledyne Isco, Inc., Lincoln, NE, USA) was installed at the outlet pipe which then drains to the stormwater pond. The autosampler was programmed to collect samples every 5 min until end of the runoff entering the pond in response to a minimum of 0.25 cm rainfall in 15 min (equivalent to rainfall intensity of 1 cm/h). Rainfall samples were collected using a clean 1 L polyethylene bucket. At the site, an ISCO 674 rain gauge (Teledyne Isco, Inc., Lincoln, NE) was used to measure rainfall. From July to November, 29 stormwater events were captured that resulted in collection of 153 stormwater runoff samples (Table 1). During this time period, 22 rainfall samples were also collected. All the collected samples were stored in a refrigerator at 4 °C and analyzed within 24 h.

A subsample of water samples was immediately (within 24 h of collection) vacuum-filtered (0.45 μm Pall Corporation, Ann Arbor, MI) in 20 mL high-density polyethyelene scintillation vials for analysis. The filtered samples were analyzed for PO_4_–P on AutoAnalyzer 3 (AA3, Seal Analytical, Mequon, WI, USA) using EPA method 365.1^69^. The unfiltered samples were analyzed for TP using the alkaline persulfate digestion method^70^ followed by PO_4_–P analysis as described above. The unfiltered samples were analyzed for TN using the alkaline persulfate digestion method^70^, followed by analysis with EPA method 353.2^71^, as described in Yang and Toor^18^. The difference between TP and PO_4_–P was determined to be other–P (combination of particulate reactive and dissolved and particulate unreactive forms). Total N data was taken from our previous study^18^ to compute TN:TP in individual storm events. Sample duplicate, reagent blank, and spikes were included in the analysis to ensure quality assurance and quality control (QA/QC). The detection limits were 0.002 mg/L for PO_4_–P and TP and 0.001 mg/L for TN.

### Statistical analysis

The one-way Analysis of Variance (ANOVA) Tukey-Kramer HSD (honest significant difference) was used to examine the significant differences (*p* < 0.05) of measured variables among various sampling months. The correlation among rainfall and stormwater runoff samples was investigated to determine the relative importance of different variables. All statistical analyses were conducted using the JMP statistical software package (JMP Pro 12, SAS Institute).

## Electronic supplementary material

Supplemenatry Information
