# Supplementary material for: Stormwater runoff driven phosphorus transport in an urban residential catchment: Implications for protecting water quality in urban watersheds
Source: Sci Rep. 2018 Aug 3;8:11681. doi: 10.1038/s41598-018-29857-x (PMC6076301; doi:10.1038/s41598-018-29857-x)
Supplement: Supplementary file 1 — Supplemenatry Information [file 41598_2018_29857_MOESM1_ESM.pdf]

Supplementary Information for

Stormwater runoff driven phosphorus transport in an urban residential catchment: Implications for protecting water quality in urban watersheds

Yun-Ya Yang and Gurpal S. Toor*

Department of Environmental Science and Technology, University of Maryland, College Park, MD 20742, USA

*Corresponding and requests for materials should be addressed to G.S.T. (email: gstoor@umd.edu)

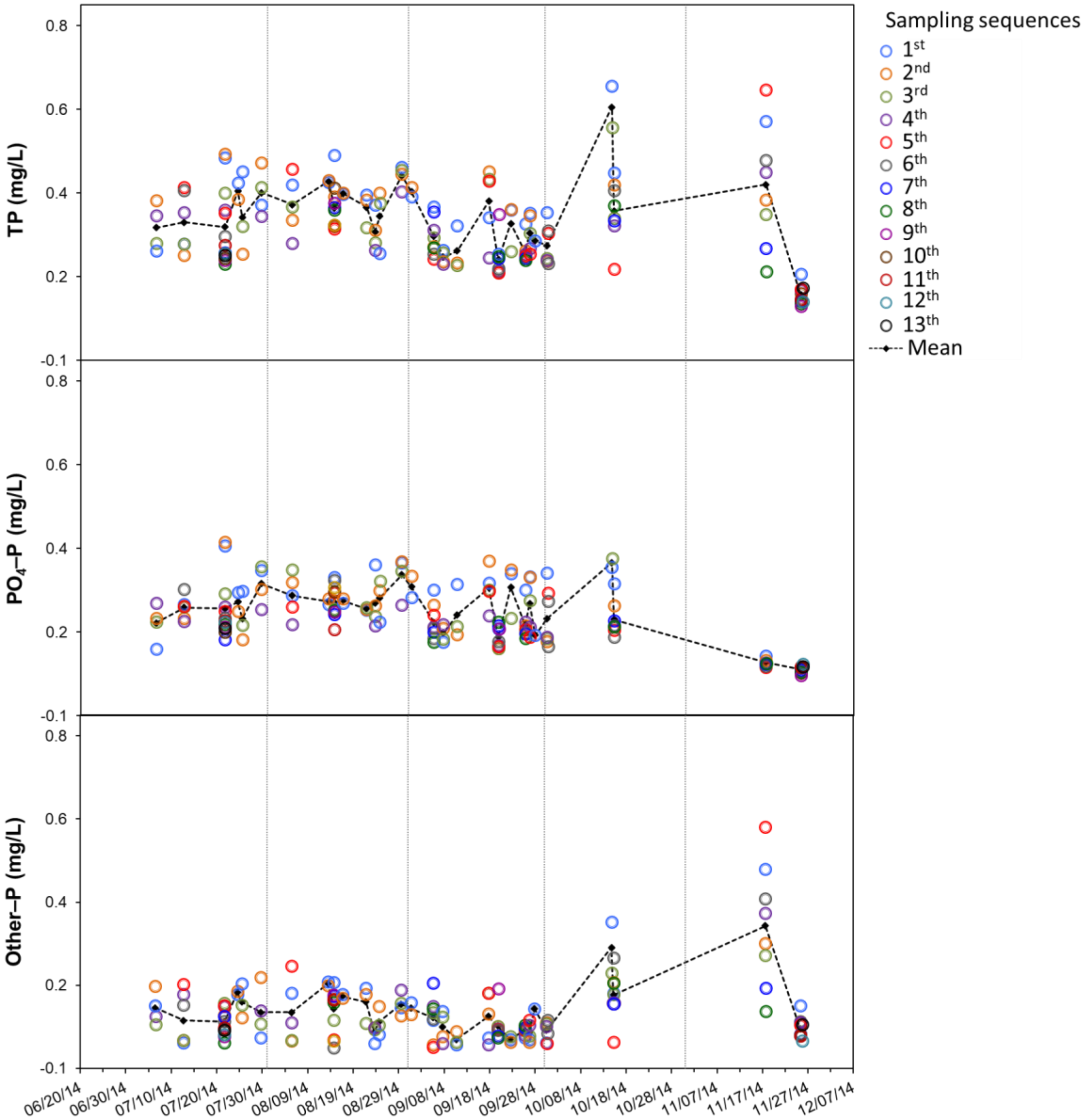

Figure S1. Concentration of TP, PO_4–P, and other-P in stormwater runoff samples (n=153) collected during 29 storm events. A sampling sequence of 1 to 13 refers to individual samples collected in 5 min intervals during each storm event. The dashed line shows change of mean values from individual events.

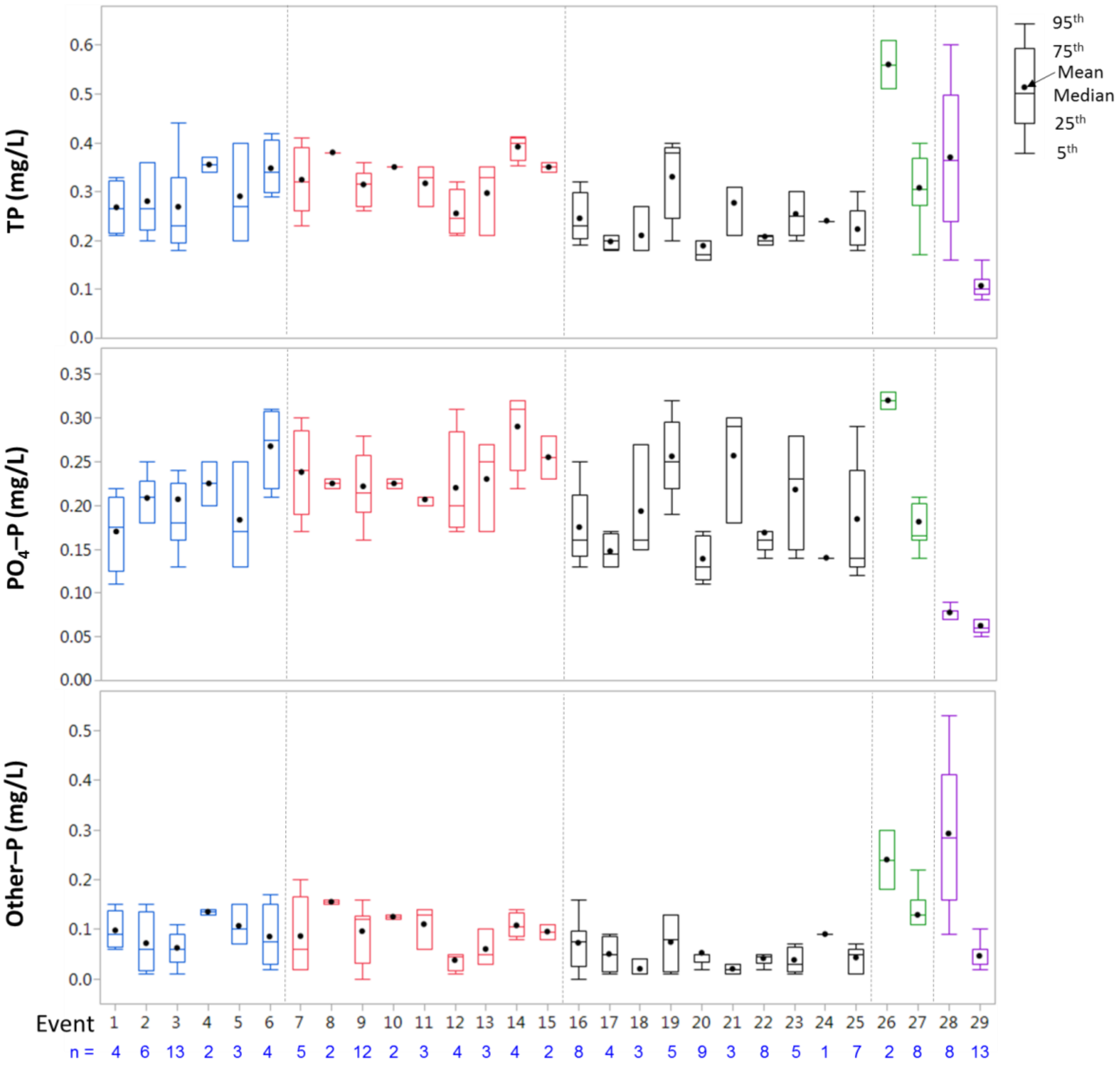

Figure S2. Concentration distributions of P forms in the stormwater runoff (n = 153) collected during 29 storm events. Event 1–6, 7–15, 16–25, 26–27, and 28–29 occurred in July, August, September, October, and November, respectively. Numbers in blue represents total number of sequential 5 min samples collected during each event.

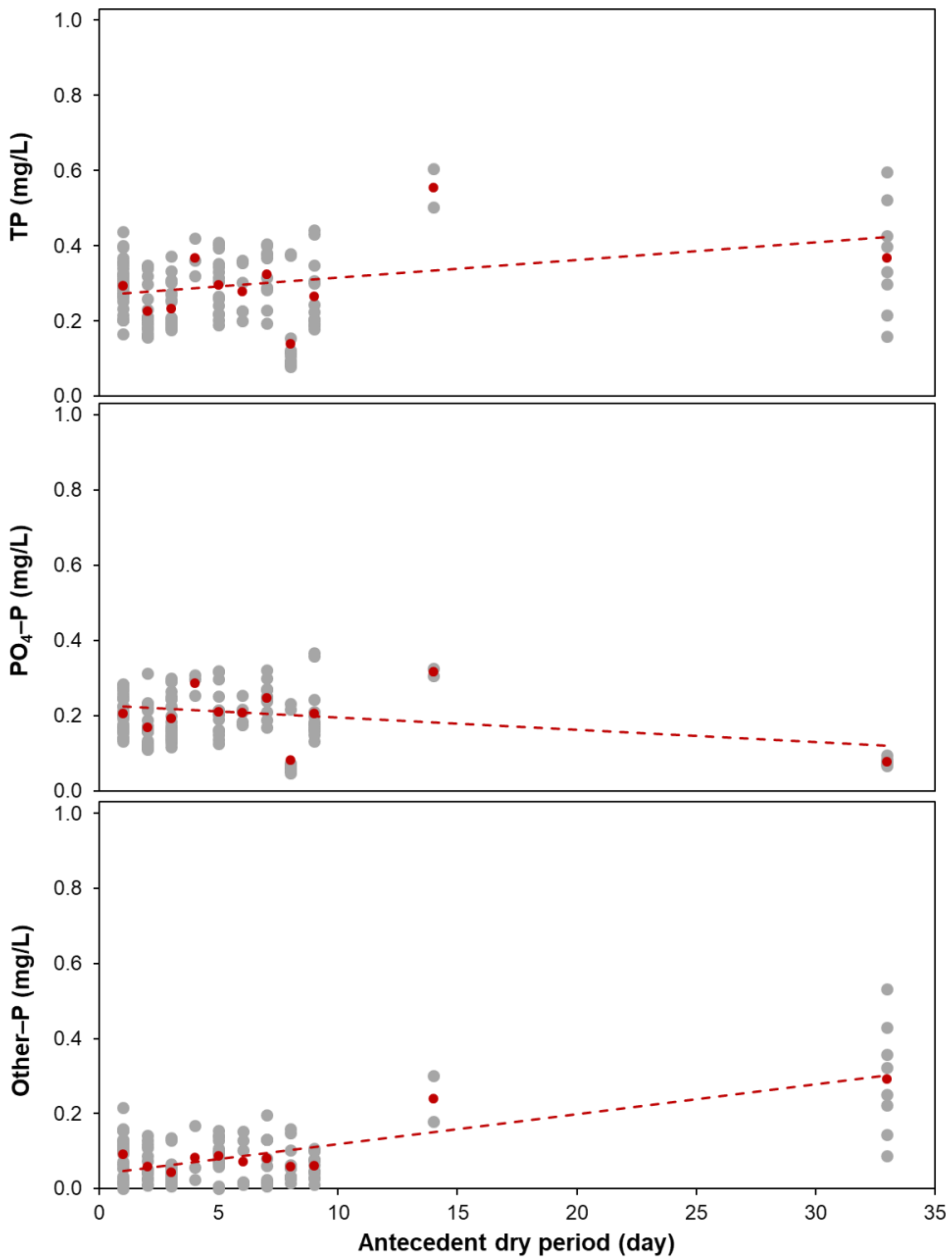

Figure S3. Concentration of P forms in the stormwater runoff (n = 153) vs antecedent dry period during 29 storm events. The red circles indicate mean concentrations for each storm event.

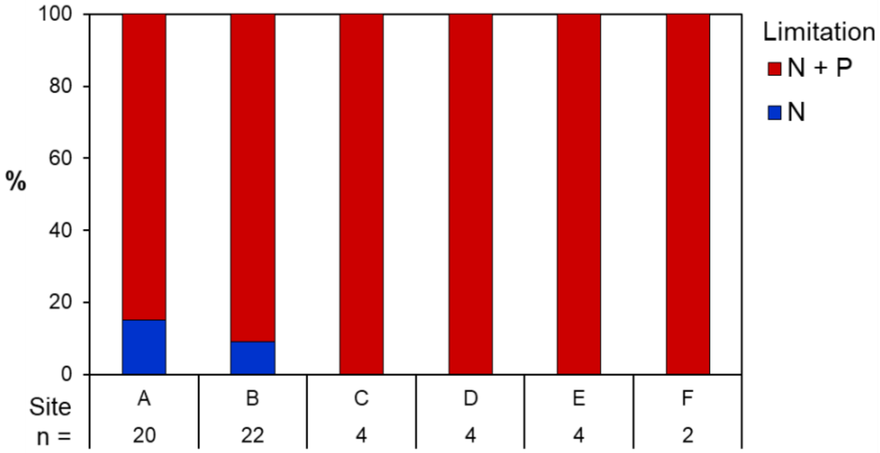

Figure S4. N and P limitation in stormwater runoff from six medium- to high-density urban residential catchments (A to F; n = 54) during August–September 2014. Strict N limitation (TN:TP ≤ 9), or N and P co-limitation (23 > TN:TP > 9), as described by Guildford and Hecky[1] and Paerl et al.[2] Concentrations of TP and TN used to calculate the mass TN:TP were obtained from Yang and Toor[3]. The detailed description of characteristics of residential catchments can be found in Yang and Toor[3].

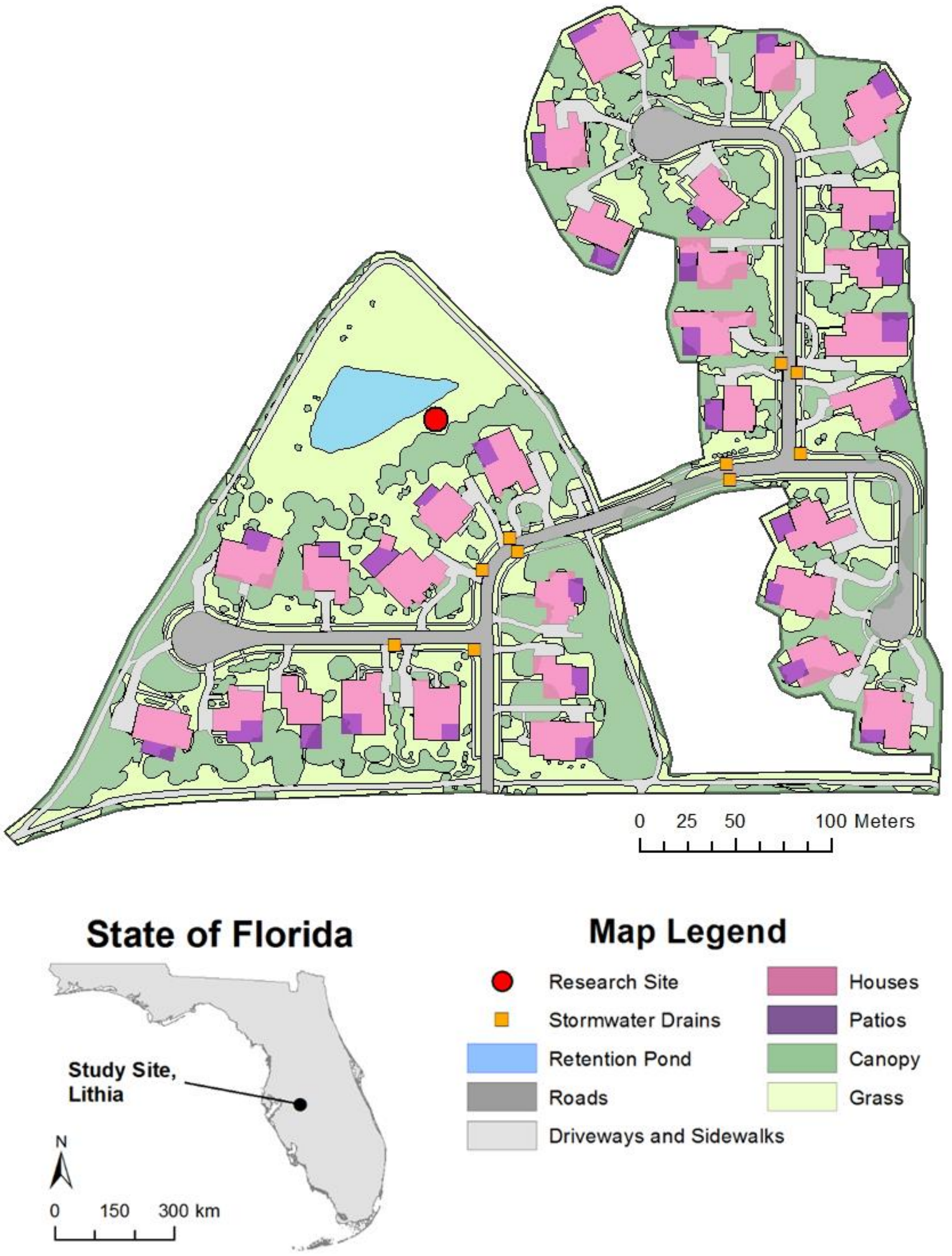

Figure S5. Location map of low-density residential catchment in Lithia, Florida, United States. Maps were generated using ArcGIS software (Version 10.3.1, Esri, http://desktop.arcgis.com/en/). The upper map was created via a map digitization method, where polygons and lines were formed based on high resolution aerial photos. All upper map layers were created by the authors. High Resolution Orthoimagery (used for reference only): USGS – Earth Resources Observation and Science (EROS) Center (2014). The lower map was generated using geographic information system (GIS) sources: Florida State Boundary: United States Census Bureau – TIGER/Line File, 1: 100,000 (1990). Map authors: Yun-Ya Yang and Stefan Kalev.

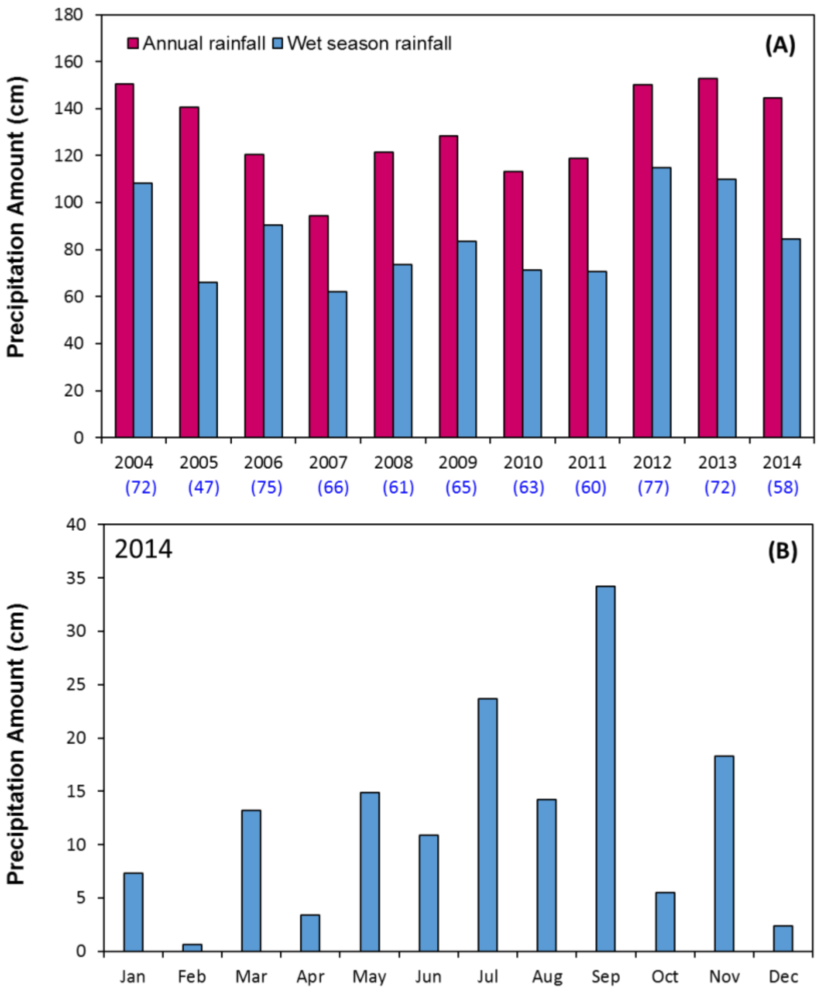

Figure S6. (A) Annual and wet season (June to September) rainfall from 2004 to 2014 and (B) Monthly rainfall during 2014. Values in the parentheses (next to the year) are percent wet season rainfall of total annual rainfall. Data from National Atmospheric Deposition Program (http://nadp.isws.illinois.edu/).

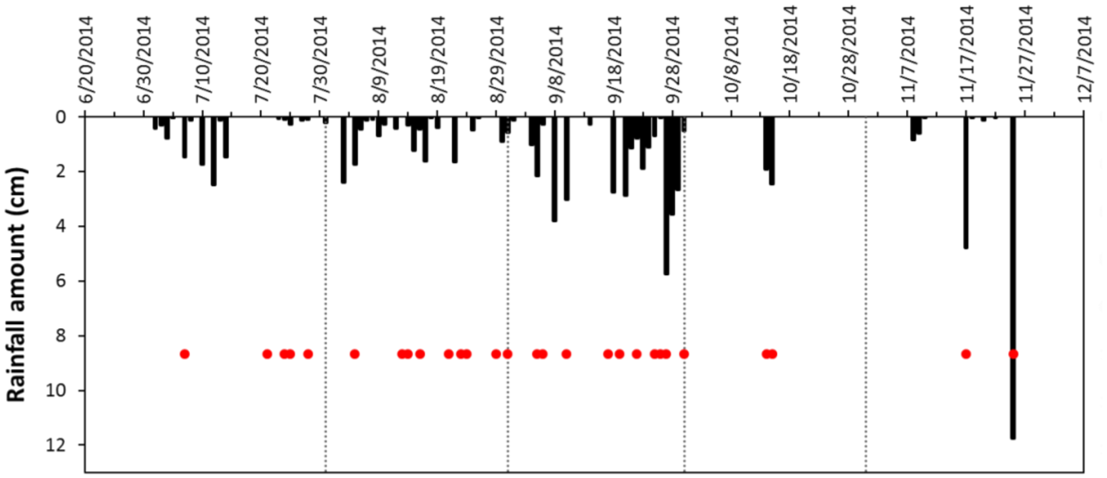

Figure S7. Temporal variability of daily rainfall from July to November 2014 at the study site. The red circles indicate 29 individual sampled storm events.

References

1. Guildford, S. J. & Hecky, R. E. Total nitrogen, total phosphorus, and nutrient limitation in lakes and oceans: Is there a common relationship? *Limnol. Oceanogr.* **45**, 1213-1223 (2000).

2. Paerl, H. W. *et al.* It takes two to tango: When and where dual nutrient (N & P) reductions are needed to protect lakes and downstream ecosystems. *Environ. Sci. Technol.* **50**, 10805-10813 (2016).

3. Yang, Y.-Y. & Toor, G. S. Sources and mechanisms of nitrate and orthophosphate transport in urban stormwater runoff from residential catchments. *Water Res.* **112**, 176-184 (2017).
